# Predictors of chronic arthralgia following chikungunya virus infection

**DOI:** 10.3205/dgkh000664

**Published:** 2026-07-17

**Authors:** Hemali Jha, Mohammad Tauseef Khan, Nida Khan, Shilpi Gupta

**Affiliations:** 1 Department of Internal Medicine, Integral Institute of Medical Sciences and Research Centre, Lucknow, Uttar Pradesh, India; 2 Department of Microbiology, Mahatma Gandhi Medical College and Hospital, Jaipur, Rajasthan, India

**Keywords:** arthralgia, chikungunya, joint pain, joint swelling, rash

## Abstract

**Objectives:**

Chikungunya is a viral illness marked by fever and joint pain. While usually self-limiting, up to 43% may develop chronic arthralgia. The objective of this study is to evaluate the frequency and predictors of prolonged joint pain (>90 d) following Chikungunya infection, with limited data available from North India.

**Methods:**

A prospective observational study was conducted at a North Indian tertiary care hospital from September 2023 to December 2023 among adults with lab-confirmed Chikungunya. A total of 56 patients were followed up to 4 months to assess joint pain duration and severity. Data were collected through clinical exams and interviews during initial workup and follow up.

**Results:**

All patients had joint pain; 23% reported joint pain lasting for more than 90 days. The ankle was the most commonly affected joint (35.7%), followed by the knee (25%) and metacarpophalangeal joints (21.4%). A comparative correlation between patients of chronic arthralgia and no chronic arthralgia showed significant association with presence of rashes, moderate to severe joint pain and swelling at the disease onset (p value <0.05).

**Conclusion:**

In the present study, moderate to severe joint pain, joint swelling, and skin rashes at disease onset were found to be the predictors of prolonged arthralgia.

## Zusammenfassung

**Zielsetzung:**

Chikungunya ist eine Viruserkrankung, die durch Fieber und Gelenkschmerzen gekennzeichnet ist. Obwohl sie in der Regel selbstlimitierend ist, können bei bis zu 43% der Betroffenen chronische Gelenkschmerzen auftreten. Ziel dieser Studie ist es, die Häufigkeit und die Prädiktoren für anhaltende Gelenkschmerzen (>90 d) nach einer Chikungunya-Infektion zu untersuchen, da aus Nordindien nur wenige Daten vorliegen.

**Methode:**

Von September 2023 bis Dezember 2023 wurde in einem nordindischen Krankenhaus der Maximalversorgung eine prospektive Beobachtungsstudie unter Erwachsenen mit laborbestätigter Chikungunya-Infektion durchgeführt. Insgesamt 56 Patienten wurden bis zu vier Monate lang nachbeobachtet, um die Dauer und den Schweregrad der Gelenkschmerzen zu beurteilen. Die Daten wurden im Rahmen klinischer Untersuchungen und Befragungen während der Erstuntersuchung und der Nachsorge erhoben.

**Ergebnisse:**

Alle Patienten litten unter Gelenkschmerzen. 23% gaben an, dass ihre Gelenkschmerzen länger als 90 d anhielten. Das Sprunggelenk war das am häufigsten betroffene Gelenk (35,7%), gefolgt vom Kniegelenk (25 %) und den Metakarpophalangealgelenken (21,4%). Ein Vergleich zwischen Patienten mit chronischen Gelenkschmerzen und solchen ohne chronische Gelenkschmerzen ergab einen signifikanten Zusammenhang mit dem Auftreten von Hautausschlägen, mäßigen bis starken Gelenkschmerzen und Schwellungen zu Beginn der Erkrankung (p<0,05).

**Schlussfolgerung:**

Es wurde festgestellt, dass mäßige bis starke Gelenkschmerzen, Gelenkschwellungen und Hautausschläge zu Beginn der Erkrankung Prädiktoren für eine anhaltende Arthralgie sind.

## Introduction

Chikungunya fever is a viral disease that has re-emerged as an important global health concern. It is a mosquito-borne febrile illness caused by an RNA alphavirus of the Togaviridae family transmitted through the bite of an infected *Aedes aegypti* mosquito [1]. The name "Chikungunya" comes from a Makonde word meaning "that which bends up," referring to the stooped posture caused by arthritic symptoms. The disease gained global attention after a 2005 epidemic on Réunion Island, France, affecting over 30% of the population [2], [3].

Chikungunya virus (CHIKV) has an incubation period of 2–7 days. In susceptible populations, attack rates may reach as high as 40 to 85%. The illness is typically self-limiting, marked by high-grade fever, polyarthralgia or arthritis, and a maculopapular rash affecting the limbs and trunk [4]. Fever generally lasts between 1 and 10 days; however, joint pain may persist for months or even years. Polyarthralgia predominantly impacts the hands and feet, leading to significant functional impairment [5]. According to a systematic review and meta-analysis, approximately 43% of patients with Chikungunya may progress to chronic phase where symptoms like arthralgia last longer than three months [6].

Although most patients recover over time, the proportion developing chronic symptoms remains uncertain, with estimates ranging from 3% to 83%. The timeline for symptom resolution is also unclear. Complete recovery from CHIKV-related arthralgia is unlikely in all cases [6], [7]. Chronic symptoms that persist after the acute phase of CHIKV infection necessitate careful management and support [8].

There is limited information regarding the prevalence of chronic arthritis in North India. Also, early identification of individuals at risk for developing chronic manifestations following CHIKV infection could optimize medical care. Thus, the present study was undertaken to evaluate the frequency of prolonged joint pain (lasting for more than 90 days) in patients with Chikungunya fever and to determine the predictors of prolonged joint pain amongst these patients.

## Methods

### Study design 

This prospective observational study was conducted at a tertiary care teaching hospital in North India. A cohort of patients with laboratory-confirmed CHIKV infection was established between September 2023 to December 2023 in individuals presenting with acute febrile illness. 

### Inclusion criteria 

Patients more than 18 years old who were seropositive on Chikungunya IgM antibody ELISA test with acute febrile illness accompanied by arthralgia/arthritis and/or a maculopapular rash.

### Exclusion criteria 

Any pre-existing chronic inflammatory arthritis condition like rheumatoid arthritis and spondyloarthropathy and patients, who were lost to follow-up before 4 months

### Data collection and follow-up 

A detailed history, demographic data collection, physical examination and joint examination for arthritis and basic lab tests were done for all the study subjects at baseline. Patients were followed up on outpatient department (OPD) basis at 2-weeks and subsequently every month till 4 months or till their joint pain disappeared. Data were collected through a face-to-face interview using a simple questionnaire format.

Duration of fever was recorded. It was categorized as short duration if it lasted for 5 days or less and long duration (if fever lasted >5 days). Joint pain severity was assessed at baseline using visual analog scale and categorized as mild and moderate/ severe [9]. The distribution of joints involved was recorded. The joint pain duration in days was recorded. It was categorized as a prolonged joint pain, if it lasted for more than or equal to 90 days. 

### Statistical analysis 

The results were presented in frequencies, mean ± standard deviation, and percentages. Patients were categorized into 2 groups, one with short duration of joint pain (<90 days) and long duration joint pain (≥90 days). Chi square test was used to compare categorical variables between the groups. The two sided P<0.05 was considered as statistically significant. All the analysis was carried out by IBM SPSS 21.0 version (Chicago, IL, Inc., USA).

### Ethical approval 

The study was approved by the Institutional Ethics Committee of the Integral Institute of Medical Sciences and Research College, Lucknow, India (IEC/IIMSR/2023/56), and written informed consent was obtained from all patients before the study. 

## Results

**Figure 1:**
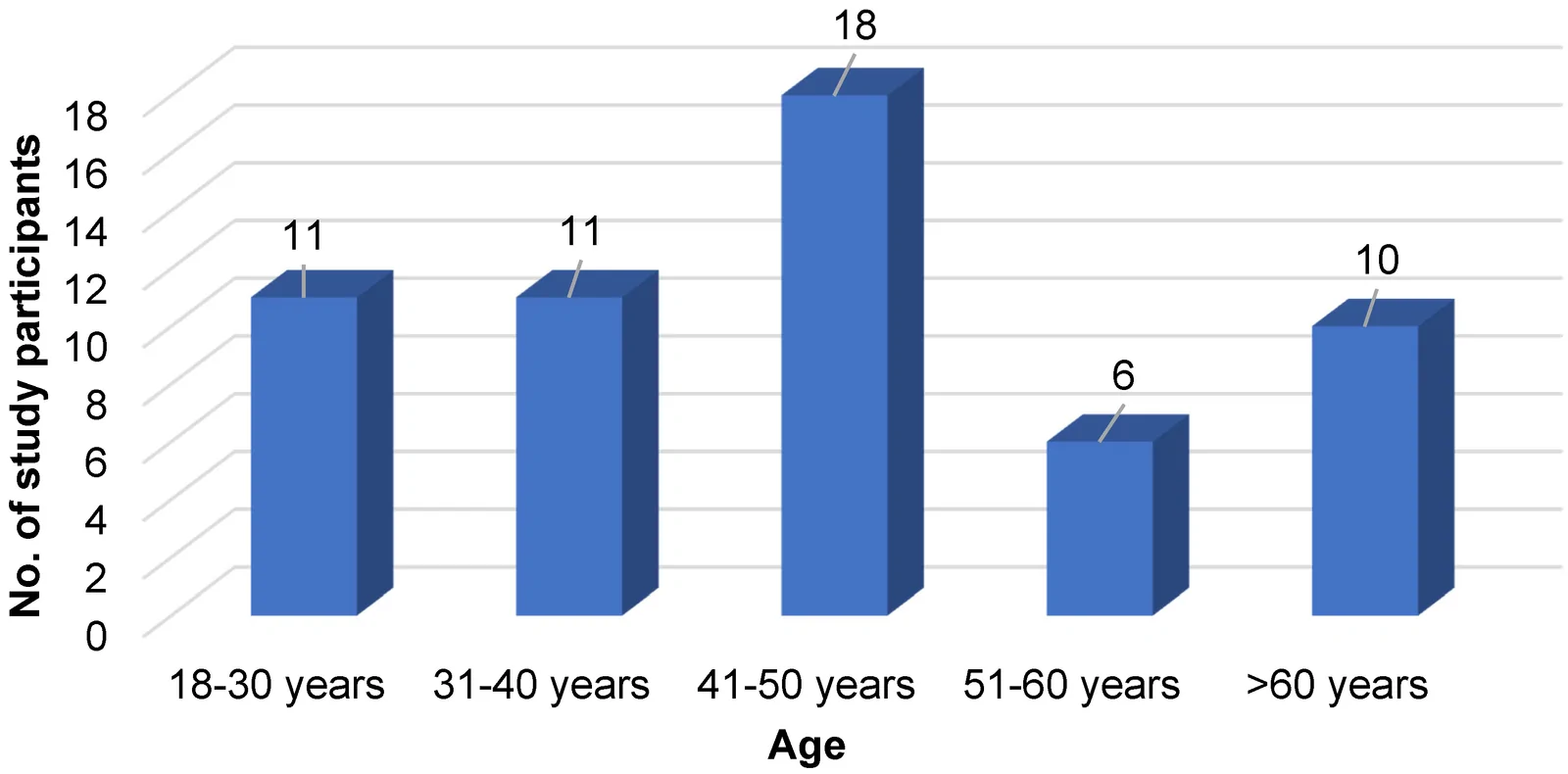
Age wise distribution of study participants (n=56)

During the study duration, 64 patients were diagnosed with CHIKV infection. Out of the 64 laboratory diagnosed chikungunya patients, follow up was successfully completed for 56 (87.5%) patients. The mean (±SD) age of the study participants was 44.2 (±14.4) years; where the minimum age was 18 years and the maximum age was 77 years with median (IQR) of 42.5 (32.5-53.5). The age distribution of study participants is shown in Figure 1 (Fig. 1) with majority of the patients were found to be aged between 41-50 years (32.1%). 

There was a female (30; 53.6%) preponderance of patients infected with Chikungunya, with a female to male ratio of 1.2:1. The baseline clinical features and laboratory findings of the patients in acute stage or during initial visit is shown in the Table 1 (Tab. 1). Of the 56 study participants, majority presented with fever of 6-10 days (57.1%), followed by fever duration between 3-5 days (35.7%). There were 3 patients with duration of fever more than 10 days accounting for 5.4% of the study participants. Also, one patient did not present with fever, but only joint pain. The mean (±SD) fever duration of the study participants was 6.8 (±3.4) days; with a maximum duration of 25 days.

All the patients complained of joint pain, with 31 patients (55.4%) having mild joint pain and remaining 25 patients (44.6%) having moderate-to-severe grade joint pain. Majority of the patients had involvement of four or less joints (93%); while polyarticular arthritis was seen in 7% patients. The proportion of patients in whom joint pain persisted for up to 15 days was 26.8%. There were 13 patients with duration of joint pain more than 90 days accounting for 23% of the study participants. The mean (±SD) duration of joint pain among the study participants was 51.0 (±41.7) days; with a maximum duration of 120 days or 4 months. The most frequently involved joints were the ankle joint (35.7%) followed by knee joint (25%) and metacarpophalangeal joint (21.4%).

The Table 2 (Tab. 2) shows comparative baseline demographic, clinical and lab characteristics in chikungunya patients with and without chronic arthritis. Among those with duration of joint pain ≥90 days, 46.2% were between 41-50 years of age and 23.1% were aged between 31-40 years. However, distribution of age according to duration of joint pain was not statistically significant (p=0.302). Also, mean age was comparable between those with joint pain <90 days and joint pain ≥90 days (p=0.294). The duration of joint pain was comparable between males and females (P value=0.511). The mean duration of fever was not found to be statistically significant between patients with joint pain <90 days and joint pain ≥90 days (P value=0.436).

## Discussion

The study was conducted to identify predictors of chronic arthralgia following CHIKV, in which participants were enrolled based on confirmed acute CHIKV infection. A total of 56 CHIKV-infected patients were followed for four months, out of which 23% developed chronic chikungunya-related arthralgia most commonly manifesting as polyarthralgia and mild functional disability. In contrast to our findings in a study conducted from North western part of India, involving 50 patients of chikungunya arthritis, 34 (68%) patients developed chronic arthralgia [10].

According to a systematic review and meta-analysis which assessed the prevalence of chronic arthralgia following chikungunya virus infection till 2015, the pooled prevalence was found to be 13.7% [11]. In similar meta-analysis the prevalence from Indian studies was reported to be 27.3% while studies from France reported 50.3% prevalence [11]. Studies from Thailand [12], and American continent documented incidence ranging from 40% to 52% [13]. These differences may be attributed to regional differences in CHIKV strains as well as host genetic factors. The pathogenesis of chronic chikungunya arthralgia (CCA) is multifaceted and remains incompletely understood. Leading hypothesis includes antigenic diversity among the circulating strains, persistence of low levels of viral nucleic acid in joint tissues for long leading to autoimmunity [14], [15].

Approximately 75% of patients who recovered from CHIKV infection without developing chronic arthralgia reported no symptoms within a month of onset. This rapid resolution may be attributed to efficient early immune responses that fac(ilitate early viral clearance and promote clinical recovery [8]. Another reason for a lower frequency of chronic chikungunya arthralgia (CCA) cases in our study could be exclusion of cases with pre-existing conditions like rheumatoid arthritis or arthropathy which in few studies were found to be associated with an increased risk of chronic chikungunya arthralgia [8], [15], [16]. Also, recent findings propose that CHIKV infection of mesenchymal stem cells (MSCs) may induce certain epigenetic modifications, potentially playing a role in the onset of chronic chikungunya arthritis [17]. Another important factor highlighted by Silva et al. [16] is the wide variation in time between the acute phase and patient follow up, combined with the inherent subjectivity of pain reporting, which also contributes to the inconsistent estimates of CCA incidence or risk.

Increasing age and female sex has been found as important predictor of development of CCA by various studies [12], [18], [19]. However, certain studies have not identified a correlation between age and disease severity or no significant gender-based differences. Though in our cohort there was female predominance for CHKV infection and also majority of cases who developed CCA. But this correlation was not found to be associated significantly. Similarly, the baseline maximum number of cases were seen in age group of 41-50 yrs but no significant correlation was attributed to increased age group range in comparison to age group less than 30 yrs.

In our study the initial severity of joint pain (based on VAS score) and presence of joint swelling at the time of presentation were found to be predictors of chronic arthralgia. The joints involved were either two or more than two joints and predominantly symmetrical. The ankle, knee, wrist and hand were the most commonly affected sites, aligning with the findings from previous studies [5], [19], [20].

Consistent with our findings, a previous study have shown that a higher number of affected joints and greater pain severity during acute phase are correlated with an increased risk of post-CHIK chronic polyarthralgia [12]. 

Another predictor of chronic arthralgia found in our study was the presence of rash during acute phase which were either maculopapular or pruritic erythematous in appearance. Several studies reported the presence of rash as one of the initial symptoms in a range from 31%–60% [5], [18], [21]. The few studies on risk factor associated with chronic arthralgia have found presence of rash in 50–60% of CCV cases however unlike our finding no significant statistical correlation was reported in their studies [12], [16]. 

### Limitations

The follow up period of only four months was small. Studies are required where follow up periods are extended at least up to a year for better understanding of evolving clinical signs and symptoms. Secondly, quantitative RT PCR was not done for association of chronic arthralgia with the viral loads.

However, our study has multiple strengths. Firstly, our sample cohort was from the same year, so possibility of genetic variability is less likely reducing the bias in comparing the cases. Apart from that, we excluded the case of any associated rheumatism or arthropathy so had an unbiased correlation of joint involvement with occurrence of chronic arthralgia.

## Conclusions

Prolonged joint pain lasting more than 90 days was observed in 13 patients (23%) with chikungunya confirmed cases. The ankle and knee joints followed by wrist joint were most commonly affected. Moderate to severe joint pain, joint swelling, and the presence of skin rashes at the disease onset were identified as predictors of persistent arthralgia lasting 90 days or longer.

## Notes

### Competing interests

The authors declare that they have no competing interests.

### Ethical approval 

The study was approved by the Institutional Ethics Committee of the Integral Institute of Medical Sciences and Research College, Lucknow, India. 

### Funding

None. 

### Generative AI statement

No artificial intelligence (AI) tools or generative AI-assisted technologies were used in the creation, drafting, or editing of this manuscript.

### Authors’ ORCIDs

- Jha H: https://orcid.org/0009-0004-8744-1569
- Khan MT: https://orcid.org/0009-0008-7579-1640
- Khan N: https://orcid.org/0009-0006-4043-0648
- Gupta S: https://orcid.org/0000-0003-0945-2524

## Figures and Tables

**Table 1:**
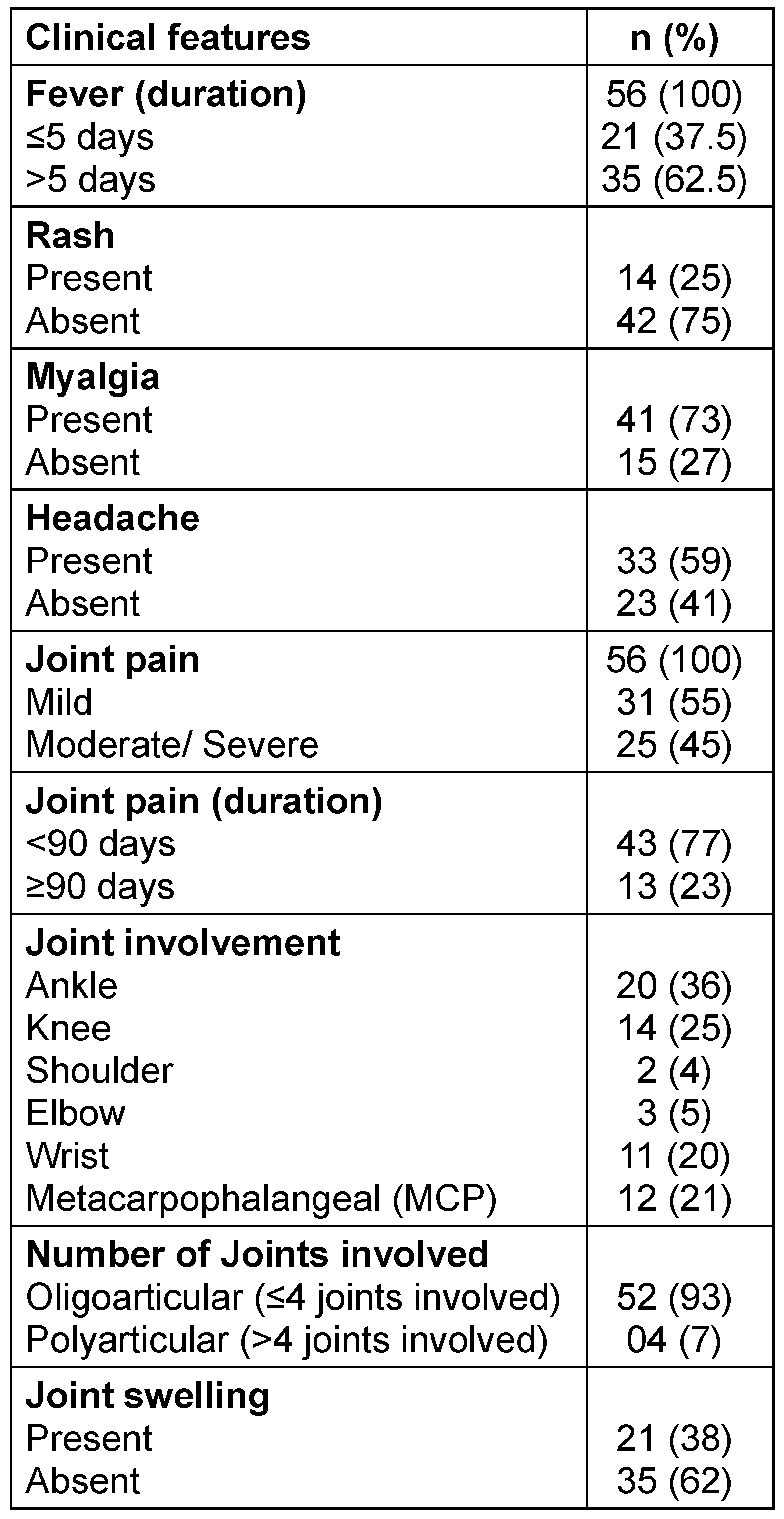
Table1: Clinical features and laboratory findings of the patients in acute stage (n=56)

**Table 2:**
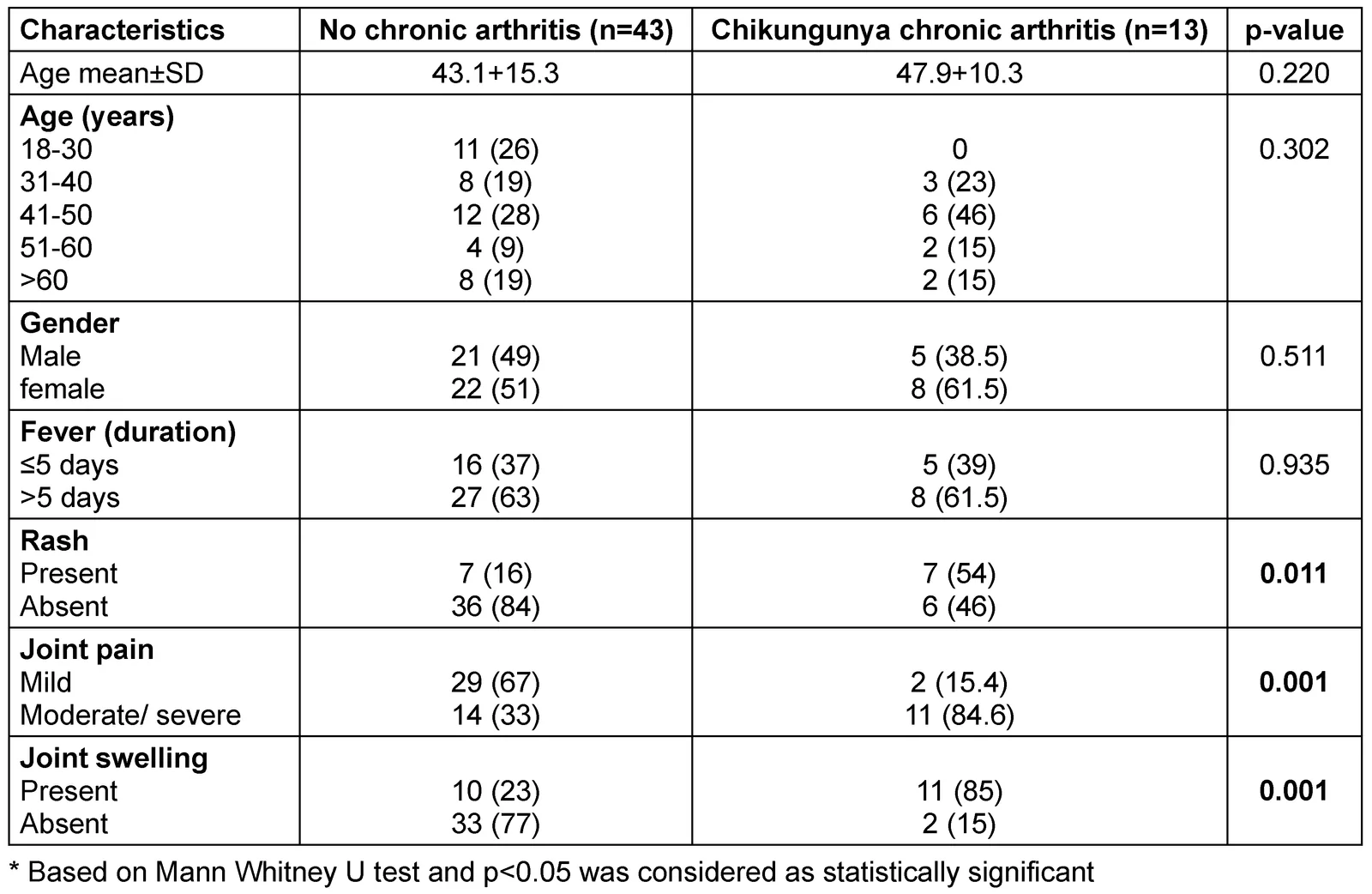
Baseline demographic and clinical characteristics in chikungunya patients with and without chronic arthritis

## References

[1] Arora R, Singh SK, Vashisht R, Gupta A, Bobdey S, Prasad R (2025). Characterizing chikungunya: a study of clinical symptoms, laboratory parameters, and therapeutics. Med J DY Patil Vidyapeeth.

[2] McSweegan E, Weaver SC, Lecuit M, Frieman M, Morrison TE, Hrynkow S (2015). The global virus network: challenging chikungunya. Antiviral Res.

[3] Ligon BL (2006). Reemergence of an unusual disease: the chikungunya epidemic. Semin Pediatr Infect Dis.

[4] Goupil BA, Mores CN (2016). A review of chikungunya virus-induced arthralgia: clinical manifestations, therapeutics, and pathogenesis. Open Rheumatol J.

[5] Hossain S, Choudhury MR, Islam MA, Hassan MM, Yeasmin S, Hossain F (2022). Post-chikungunya arthritis: a longitudinal study in a tertiary care hospital in Bangladesh. Trop Med Health.

[6] Paixão ES, Rodrigues LC, Costa MDCN, Itaparica M, Barreto F, Gérardin P (2018). Chikungunya chronic disease: a systematic review and meta-analysis. Trans R Soc Trop Med Hyg.

[7] O'Driscoll M, Salje H, Chang AY, Watson H (2021). Arthralgia resolution rate following chikungunya virus infection. Int J Infect Dis.

[8] Moraes de L, Cerqueira-Silva T, Nobrega V, Akrami K, Santos LA, Orge C (2020). A clinical scoring system to predict long-term arthralgia in chikungunya disease: a cohort study. PLoS Negl Trop Dis.

[9] Reed MD, Van Nostran W (2014). Assessing pain intensity with the visual analog scale: a plea for uniformity. J Clin Pharmacol.

[10] Gauri LA, Thaned A, Fatima Q, Yadav H, Singh A, Jaipal HP (2016). Clinical spectrum of chikungunya in Bikaner (North Western India) in 2006 and follow up of patients for five years. J Assoc Physicians India.

[11] Rodríguez-Morales AJ, Cardona-Ospina JA, Urbano-Garzón SF, Hurtado-Zapata S (2016). Prevalence of post-chikungunya infection chronic inflammatory arthritis: a systematic review and meta-analysis. Arthritis Care Res (Hoboken).

[12] Yodtaweepornanan P, Pongsittisak W, Satpanich P (2023). Incidence and factors associated with chronic chikungunya arthritis following chikungunya virus infection. Trop Med Int Health.

[13] Edington F, Varjão D, Melo P (2018). Incidence of articular pain and arthritis after chikungunya fever in the Americas: a systematic review of the literature and meta-analysis. Joint Bone Spine.

[14] Dupuis-Maguiraga L, Noret M, Brun S, Le Grand R, Gras G, Roques P (2012). Chikungunya disease: infection-associated markers from the acute to the chronic phase of arbovirus-induced arthralgia. PLoS Negl Trop Dis.

[15] Sissoko D, Malvy D, Ezzedine K, Renault P, Moscetti F, Ledrans M (2009). Post-epidemic chikungunya disease on Reunion Island: course of rheumatic manifestations and associated factors over a 15-month period. PLoS Negl Trop Dis.

[16] Silva MMO, Kikuti M, Anjos RO, Portilho MM, Santos VC, Gonçalves TSF (2021). Risk of chronic arthralgia and impact of pain on daily activities in a cohort of patients with chikungunya virus infection from Brazil. Int J Infect Dis.

[17] Amaral JK, Bingham CO, Taylor PC, Vilá LM, Weinblatt ME, Schoen RT (2023). Pathogenesis of chronic chikungunya arthritis: resemblances and links with rheumatoid arthritis. Travel Med Infect Dis.

[18] Heath CJ, Lowther J, Noël TP, Mark-George I, Boothroyd DB, Mitchell G (2018). The identification of risk factors for chronic chikungunya arthralgia in Grenada, West Indies: a cross-sectional cohort study. Open Forum Infect Dis.

[19] Ramachandran V, Kaur P, Kanagasabai K, Vadivoo S, Murhekar MV (2014). Persistent arthralgia among chikungunya patients and associated risk factors in Chennai, South India. J Postgrad Med.

[20] Schilte C, Staikowsky F, Couderc T, Madec Y, Carpentier F, Kassab S (2013). Chikungunya virus-associated long-term arthralgia: a 36-month prospective longitudinal study. PLoS Negl Trop Dis.

[21] Suryawanshi SD, Dube AH, Khadse RK, Jalgaonkar SV, Sathe PS, Zawar SD (2009). Clinical profile of chikungunya fever in patients in a tertiary care centre in Maharashtra, India. Indian J Med Res.

